# Henoch-Schönlein Purpura /IgA Vasculitis Complicated by Coronary Artery Aneurysm: A Case Report and Literature Review

**DOI:** 10.3389/fped.2021.781106

**Published:** 2022-02-03

**Authors:** Zhijuan Kang, Wentao Wu, Mai Xun, Yunfeng Ding, Zhihui Li

**Affiliations:** ^1^Department of Nephrology, Rheumatology and Immunology, Hunan Children's Hospital, Changsha, China; ^2^The School of Pediatrics, Hengyang Medical School, University of South China, Changsha, China

**Keywords:** Henoch-Schönlein purpura, children, renal involvement, coronary artery dilation, cardiac involvement, IgA vasculitis

## Abstract

Henoch-Schönlein purpura (HSP)/ IgA vasculitis (IgAV) is the most common form of systemic vasculitis in children and often involves the skin, gastrointestinal tract, joints, and kidneys, though cardiac involvement rarely occurs. We report on a 6-year-old male child with HSP/IgAV who had renal and cardiac involvement at the initial stage of the disease and in whom we found an extremely rare coronary artery aneurysm. After administration of glucocorticoid combined with mycophenolate mofetil, the renal involvement improved, but the coronary artery aneurysm remained. Pursuant to this case, we retrieved information on other cases of HSP/IgAV complicated with cardiac involvement from the PubMed database, and excluded cases of cardiac involvement accompanied by Kawasaki disease, polyarteritis nodosa, rheumatic fever, Takayasu arteritis, systemic lupus erythematosus, poststreptococcal glomerulonephritis, or sepsis. We then analyzed gender, age, cardiac involvement, renal involvement, treatment, and prognoses. To date, 24 cases of HSP/IgAV complicated with cardiac involvement have been reported. Among them, there were 22 male and 2 female patients, with the onset age ranging from 3 to 71 years old. A total of 10 children (including the child we examined) and 14 adults were identified, and 17 patients (70.8%) had HSP/IgAV complicated with renal involvement. The majority of patients were treated with glucocorticoid and/or immunosuppressants or biological agents, 4 patients died (16.7%), 8 patients were completely relieved (33.3%), and 3 patients had unknown prognoses. This article suggests that HSP/IgAV complicated with cardiac involvement may result in a poor prognosis and early treatment may therefore be essential. Our case revealed that glucocorticoid does not prevent the occurrence of renal and cardiac involvement in HSP/IgAV patients. If HSP/IgAV is complicated with coronary artery dilation, the therapeutic effect of glucocorticoid combined with immunosuppressants is not satisfactory, and early administration of biological agents or IVIG may be an effective therapeutic regimen.

## Introduction

Henoch-Schönlein purpura (HSP) is a leukocyteclastic vasculitis characterized by the deposition of immunoglobulin A (IgA)-based immune complexes on the walls of small blood vessels (1). The incidence rate of HSP is estimated to be 10–20 cases per 100,000 children, and 90% of cases are children less than ten years of age (2, 3). Several factors can trigger HSP, such as infection, medications, and environmental factors (3). Although the pathogenesis of HSP is still unclear, it has been found that there is a close relationship between HSP and IgA, and for this reason, HSP is also called IgA vasculitis (IgAV).

In addition to palpable purpura, about 75% of patients with HSP/IgAV have arthritis; 50–75% have gastrointestinal symptoms; and the incidence of renal involvement is about 40–50% (3). In recent years, a number of scholars have concentrated on HSP/IgAV occurring with other rare complications, such as pulmonary hemorrhage, orchitis, cerebral hemorrhage, stenotic ureteritis, and pancreatitis, leading to a poor prognosis (4–8). However, HSP/IgAV complicated with cardiac involvement is quite rare. At present, some cases of HSP/IgAV patients whose conditions are complicated with myocardial damage, myocardial infarction, atrial and ventricular dilation, arrhythmia, atrioventricular block, or thrombosis have been reported. However, there are few reports of HSP complicated with coronary dilation. Herein, we discuss the case of a 6-year-old HSP/IgAV patient with renal involvement and a coronary artery aneurysm. After treatment with glucocorticoid and immunosuppressant for more than 1 year, proteinuria disappeared, while coronary artery aneurysm still existed. Previously reported HSP/IgAV patients with cardiac involvement are reviewed as well.

## Case Presentation

A 6-year-old male child was admitted to our hospital on September 3, 2019 because of hemorrhagic rash of lower limbs with swelling and pain in both knee joints for 10 days, proteinuria for 3 days, and chest tightness for 1 day. Ten days before his admission, he had hemorrhagic rash of lower limbs without obvious inducement, symmetrical distribution, no itching, swelling, bilateral knee pain, and limited movement. The local hospital diagnosed him with HSP/IgAV, and injected cetirizine, dipyridamole, and methylprednisolone (2 mg/kg/d) intravenously. The joint swelling and pain improved and the rash was gradually subsided. Three days before his admission to our hospital, urine examination performed in that local hospital showed protein 2+ and occult blood 3+. At this time, the glucocorticoid has been changed to prednisone and reduced to 0.5 mg/kg/d. One day before his admission to our hospital, the patient developed chest tightness, crushing pain in the precordial area, nausea and vomiting. Thus, he was referred to our hospital for treatment. No fever, conjunctival congestion, chapped lips, or redness and swelling at the end of fingers and toes could be detected. The patient was previously in good health and had no family history of a special disease.

When the patient was admitted to our hospital, his body temperature was 36.3°C (axillary temperature); his heart rate was 105 beats/min; his respiratory rate was 18 beats/min; his blood pressure was 97/65 mmHg; and his body weight was 18 kg. After physical examination, scattered old rashes were found on the lower limbs, and no obvious positive signs were identified.

The results of blood work performed after admission were as follows. The blood routine test showed that the number of white blood cells (WBC) and platelets (PLT) was elevated; the erythrocyte sedimentation (ESR) rate increased; the results of blood biochemistry revealed that the levels of aspartate aminotransferase (AST), creatine kinase (CK), creatine kinase isoenzyme (CK-MB), lactate dehydrogenase (LDH), and troponin I elevated; antineutrophil (ANA) spectrum, ANA cytoplasmic (ANCA), and vasculitis-related antibodies were negative; the result of etiological test was negative; and the urine test showed heavy proteinuria and microscopic hematuria (Table 1).

**Table 1:** Laboratory examination results of the HSP/IgAV child after hospitalization.

Blood routine test		Pathogenic examination	
White blood cell (×10^9^/L)	24.77	PPD-IgG/IgM	Negative
Neutrophils ratio (%)	0.680	SPOT-TB test	Negative
Lymphocyte ratio (%)	0.253	Mp-Ab	Negative
Hemoglobin (g/L)	143	HIV-Ab	Negative
Platelet (×10^9^/L)	640	CMV-DNA(0–400 Copies/mL)	<400
Red blood cell (×10^12^/L)	5.09	EBV-DNA(0–400 Copies/mL)	<400
Blood biochemical test		ASO(0–100 IU/mL)	<25
ALB (35–55 g/L)	38.8	Autoimmune antibody	
AST (0–40 IU/L)	119.1	Anti-nuclear antibodies (ANAs)	Negative
ALT (0–40 IU/L)	45.8	P-ANCA	Negative
Creatinine(μmol/L)	33.0	C-ANCA	Negative
Urea nitrogen(mmol/L)	4.32	ACA IgA/ IgG/ IgG	Negative
CK (38–174 U/L)	976	GBM/MPO/PR3-IgG	Negative
CK-MB (0–24 IU/L)	71.6	Coagulation function tests	
LDH (0–450 IU/L)	965	PT (10–14s)	12.9
Troponin I (<0.01 ug/mL)	9.77	INR (0.8–1.5)	0.99
Acute-phase reactants		APTT (28–48s)	30.4
C-reactive protein (0–8 mg/L)	<0.5	AT3 (80–120%)	126
ESR (0–15 mm/h)	29	TT (14–20s)	16.6
Immunoglobulins		FIB (170–450 mg/dL)	226
Ig A (0.14–1.38 g/L)	2.01	D-Dimer (0–0.55ug/mL)	0.38
Ig E (<90 IU/mL)	2,780	FDP (0–5 ug/mL)	2.38
Ig G (3.6–10.6 g/L)	7.69	Urine analysis	
Immunoglobulin M (0.38–1.44 g/L)	1.50	Proteinuria (Negative)	3+
Complements		Urine red blood cell count (0–22.8/ul)	143.5
C3(0.79–1.52 g/L)	1.02	Proteinuria-24 h (0–150 mg/24h)	1,210
C4(0.16–0.38 g/L)	0.16	urine protein/creatinine ratio (mg/mg)	3.35

*Mp-Ab, Mycoplasma Pneumoniae Antibodies; TB, tubercle bacillus; ANCA, anti-neutrophilic cytoplasmic antibodies; PR3-IgG, proteinase 3-IgG; MPO-IgG, myeloperoxidase-IgG; GBM-IgG, glomerular basal membrane-IgG; ACA, anti-cardiolipin antibody*.

**Figure 1:**
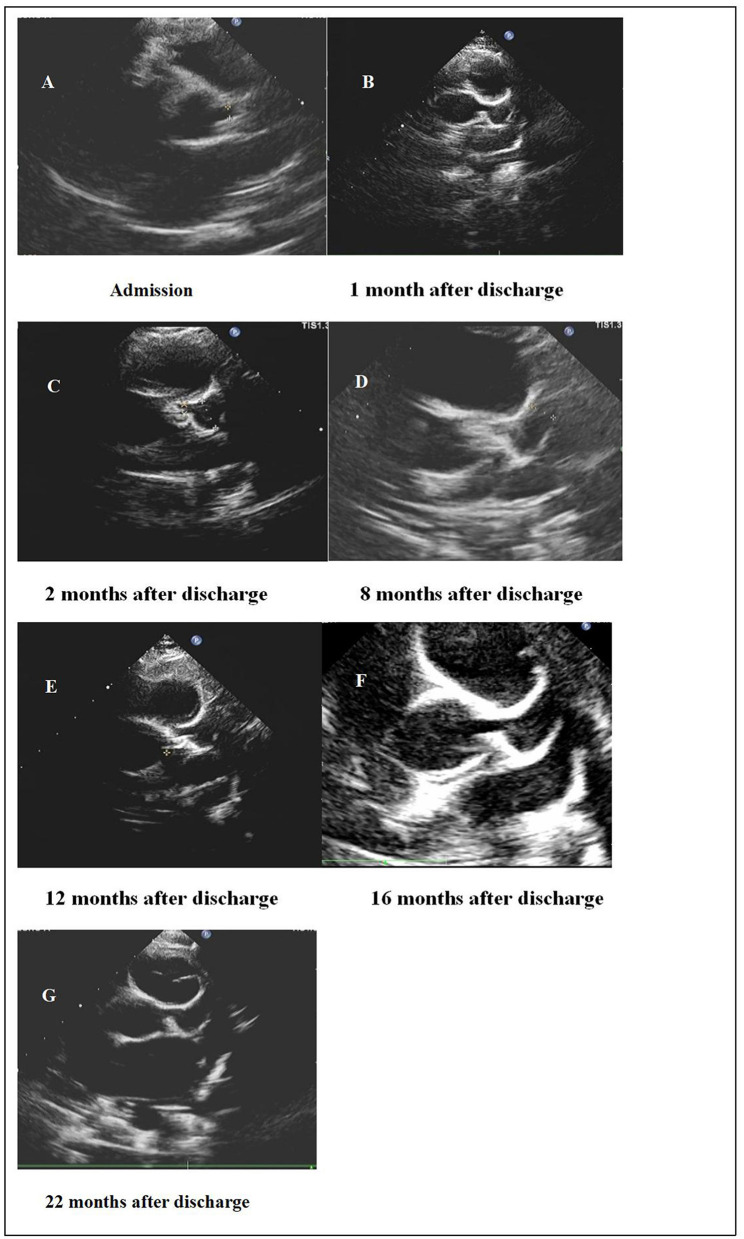
Dilation of the left coronary artery showed by cardiac color Doppler ultrasound.

The patient had chest tightness and crushing pain in the precordial area. The electrocardiogram (ECG) showed normal sinus rhythm and left axis deviation. The 24-h dynamic ECG displayed sinus tachycardia, occasional atrial premature beats (one in the whole day), and abnormal Q wave (II, III, aVF, V5 and V6 were obvious). Cardiac color Doppler ultrasound showed: bilateral coronary artery dilatation grade I [left main trunk, 3.6 mm (Z-value + 3.2);right main trunk, 3.0 mm (Z-value + 2.6)], thickening of the ventricular wall, mild mitral regurgitation and tricuspid regurgitation, and normal range of left ventricular systolic function (Figure 1A). Color Doppler ultrasound of kidneys revealed increased echogenicity of the kidney parenchyma, and there was no obvious abnormality in the color Doppler ultrasound of arteries of the abdomen, carotid arteries, or deep arteries of the limbs.

**Figure 2:**
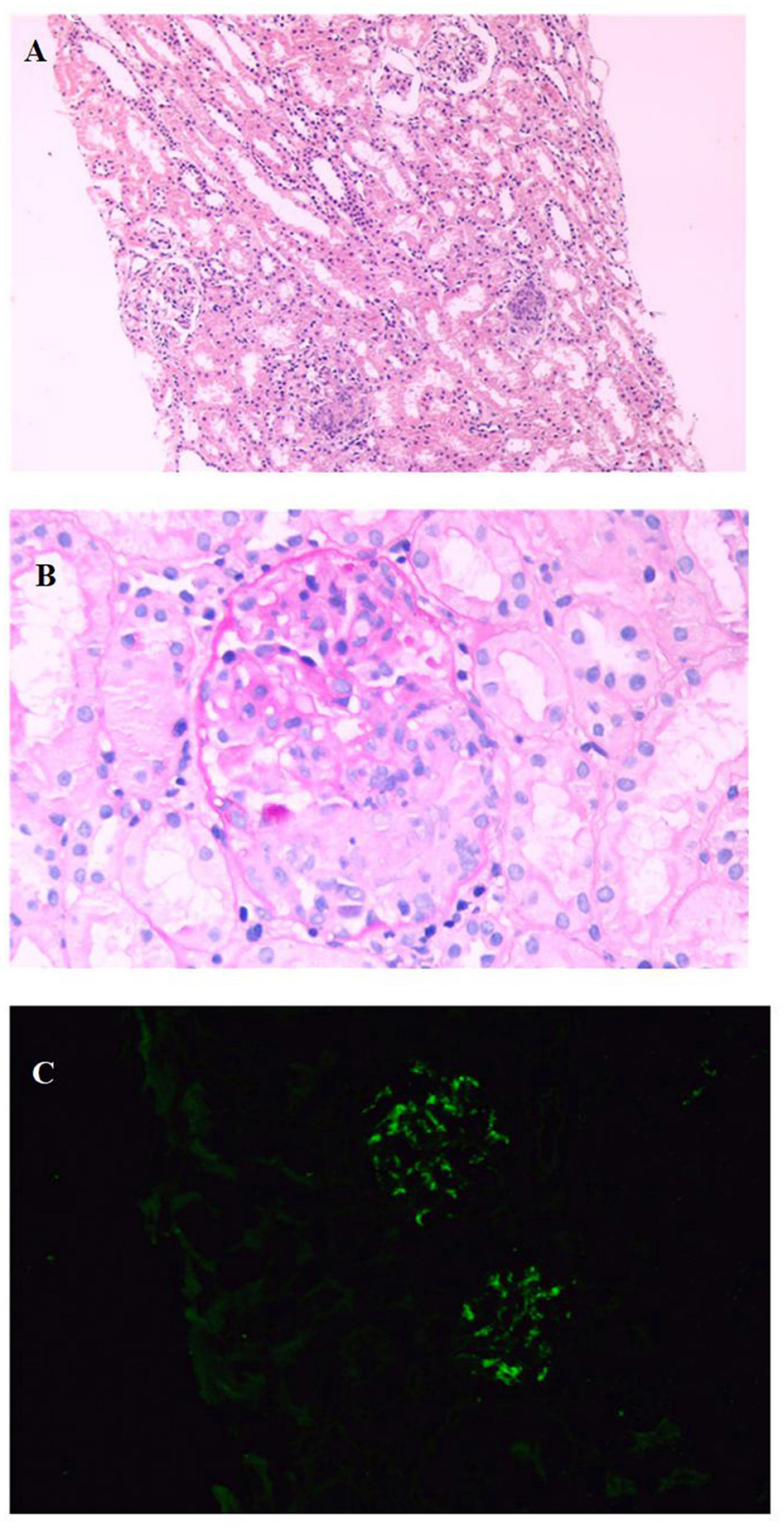
Renal biopsy shows: **(A)**. The number of cells in the glomerulus increased, focal and segmental hyperplasia of mesangial cells and stroma, and multiple crescents (HE, X100); **(B)**. Cellular crescents (PAS, X400); **(C)**. IgA deposited in glomerular mesangial area (IF, X400).

The patient was complicated with hematuria and proteinuria, and Henoch-Schönlein purpura nephritis (HSPN)/ IgA vasculitis with nephritis (IgAVN) was considered. Renal biopsy by immunofluorescence showed that IgA++ was deposited in the glomerular mesangial area. Under a light microscope, 4 crescents were found in 16 glomeruli, as well as focal and segmental hyperplasia of the mesangial cells and matrix. Under an electron microscope, there were more electron-dense deposits in the glomerulus, and there was vacuoles and granular degeneration of renal tubular epithelial cells, and segmental fusion of the foot process. Diagnosis: HSPN/ IgAVN IIIa (Figure 2).

After the onset of the disease, the local hospital diagnosed HSP/ IgAV according to the typical clinical symptoms (rash, joint swelling, and pain), and the patient was treated with glucocorticoid. Later, the patient was transferred to our hospital for treatment, because of renal involvement (hematuria and proteinuria) and cardiac involvement (chest tightness and crushing precordial pain). After admission to our hospital, the testing of the patient indicated myocardial injury, and cardiac color Doppler ultrasound showed coronary artery dilatation. As HSP/ IgAV is rarely complicated with coronary artery dilatation, other vasculitis diseases, such as Kawasaki disease, Takayasu arteritis, and polyarteritis nodosa, were considered in the differential diagnosis and treatment processes. However, the patient had no clinical symptoms of Kawasaki disease, and no other blood vessels were found to be involved by vascular color Doppler ultrasound. Therefore, we considered the coronary artery dilatation caused by HSP/ IgAV based on the clinical symptoms and the results of the examination of the patient.

After admission to our hospital, as the patient had heavy proteinuria and liver damage, methylprednisolone (28 mg/d) and aspirin (50 mg/d) were administered orally, and cardioprotective therapy was given as well. On September 20, 2019, HSPN/IgAVN IIIa was diagnosed due to renal pathology, and mycophenolate mofetil (0.25g q12h/d) was given orally. When discharged, the blood tests were re-checked as follows: WBC, 20.19 × 10^9^/L; hemoglobin (Hb) level, 130 g/L; PLT, 468 × 10^9^/L; CK, 25.20 U/L (38–174); CK-MB, 18.70 IU/L (0–24); LDH, 224.0 IU/L (0–450); and troponin I,0.43 μg/ml (< 0.01). His liver function had returned to normal, and 24-h urine protein was 1,000 mg (0–150). The child was regular follow-up after discharge (Table 2 and Figure 1).

**Table 2:** Follow-up of proteinuria, troponin I, and cardiac color Doppler ultrasound of the HSP/IgAV child after discharge.

**Item**		**Month (s) after discharge**					
	**During hospitalization**	**1**	**2**	**8**	**12**	**16**	**22**
24-h urinary protein quantification (mg)	1,210	260	230	190	90	70	
Troponin I (μg/mL)	9.77	<0.01	-	-	-	-	-
**Left coronary artery**							
Backbone (Z-value: score)	3.6 mm (+3.2)	3.7 mm (+3.32)	3.6 mm (+2.98)	3.9 mm (+3.82)	3.5 mm (+2.62)	3.3 mm (+1.9)	3.7 mm (+3.0)
Anterior descending branch (Z-value: score)	-	6.8 mm (+16.13)	11 mm (+30.68)	6.7 mm (+15.67)	11 mm (+30.63)	9.6 mm (+25.7)	10.6 mm (+29.03)
**Right coronary artery**							
Backbone (Z-value: score)	3.0 mm (+2.6)	3.3 mm (+3.36)	2.8 mm	4.1 mm (+5.7)	2.6 mm (+1.20)	2.6 mm (+1.20)	2.7 mm (+1.37)
Distal wider inner diameter (Z-value: score)	-	4.3 mm (+6.37)	4.2 mm (+6.051)	-	-	-	-

One month after discharge, the results of the blood test were as follows: WBC, 16.81 × 10^9^/L; Hb, 141.00 g/L; PLT, 421 × 10^9^/L; C-reactive protein (CRP) level, 0.50 mg/L (0–8); ESR, 2 mm/h (0–15); 24-h urine protein, 260 mg (0–150); and troponin I < 0.01 μg/ml (< 0.01). In addition, his myocardial enzyme level and liver function were normal. These results suggested that his HSP/IgAV had improved, but his coronary artery dilatation was worse than before. The cardiac color Doppler ultrasound showed that the left coronary artery aneurysm (grade III), the right coronary artery dilatation (grade II), and the left heart systolic function were normal (Figure 1B). The patient's therapeutic regimen was adjusted as follows: the glucocorticoid was gradually reduced, while warfarin (0.6 mg/d) was added according to the patient's prothrombin time-international normalized ratio (PT-INR), and the dose of mycophenolate mofetil and aspirin did not change. The patient was regularly visited in order to adjust his therapeutic regimen, and prednisone and mycophenolate mofetil were discontinued on January, and December, 2020, respectively. The last cardiac color Doppler ultrasound were performed on June 27, 2021 and it showed that the anterior descending branch of the left coronary artery had expanded, and that the right coronary artery had returned to normal (Figure 1G). At present, aspirin (75 mg/d) and warfarin (1.8 mg/d) are given orally (September, 2021).

## Literature Review

**Figure 3:**
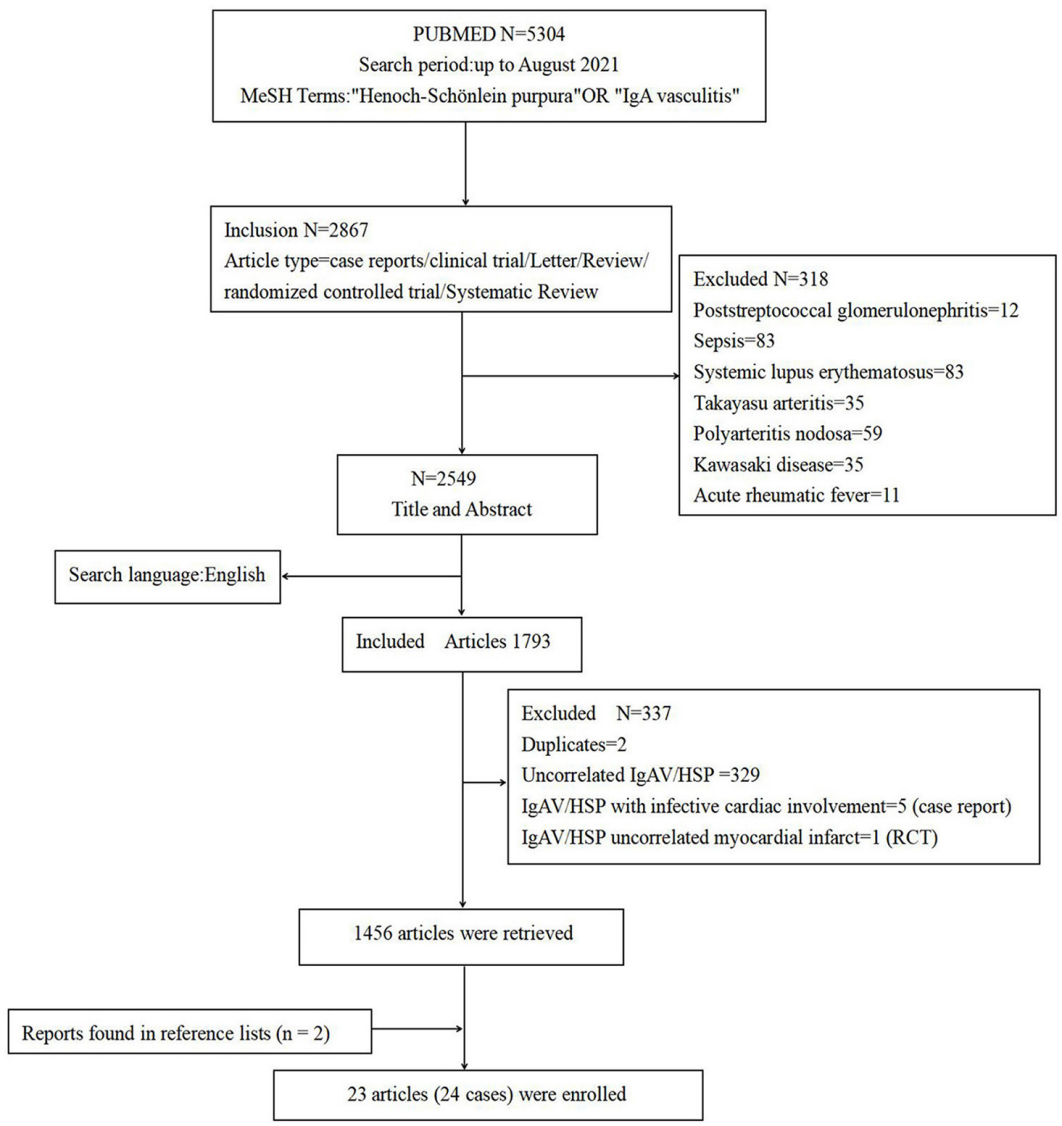
Flowchart of the literature search.

In order to understand cardiac involvement in HSP/IgAV, we searched the PubMed database from inception until August, 2021 using the keywords “Henoch-Schönlein purpura” or “IgA vasculitis,” and limited the search language to English. We included articles related to HSP/IgAV complicated with cardiac involvement, but excluded other diseasessuch as rheumatic fever, Kawasaki disease, Takayasu arteritis, polyarteritis nodosa, systemic lupus erythematosus, poststreptococcal glomerulonephritis and sepsis (Figure 3).

In total we retrieved 1,458 related articles (2 articles in reference lists). We found 23 articles (for a total of 24 cases including our case) reported definite cardiac involvement caused by HSP/IgAV. These comprised 22 male and 2 female patients with onset ages of 3 to 71 years old. A total of 17 cases (70.8%) had renal involvement; 4 cases (16.7%) resulted in death; 8 cases (33.3%) were completely cured; and 3 cases had unknown outcomes (Table 3).

**Table 3:** Comparison of reported pediatric and adult HSP/IgAV patients with cardiac involvement.

	**Children with HSP**	**Adults with HSP**
	**complicated with**	**complicated with**
	**cardiac involvement**	**cardiac involvement**
	**(*n* = 10)**	**(*n* = 14)**
Age of onset (y)	9.5 (7.5–13.0)	60.0 (21.0–63.0)
Male (*n*, %)	8 (80)	14 (100)
Renal involvement (*n*, %)	6 (60)	11 (78.5)
Death (*n*, %)	1 (10)	3 (21.4)
Complete remission (*n*, %)	3 (30)	5 (35.7)
Treatment record (*n*)	9	12
glucocorticoid use (*n*, %)	7 (77.8)	10 (83.3)
Immunosuppressant use (*n*, %)	3(33.3)	3 (25)
Use of biological agents (*n*, %)	1 (11.1)	1 (8.3)

Analysis of HSP/IgAV complicated with cardiac involvement in children (age <18 years) and adults showed that there were 14 cases in adults (9–21) and 10 cases (including the child we examined) in children (Table 4). We further analyzed gender, age, cardiac involvement, renal involvement, treatment, and prognoses. We found that the age distribution of 10 HSP/IgAV children with cardiac involvement was between 3 and 17 years old. Of these cases 8 (80%) were male, 2 (20%) were female, 6 (60%) were complicated with renal involvement, and 9 had clear therapeutic regimens. After treatment, 1 patient (10%) died with myocardial necrosis, and 1 case had an unknown outcome. There were 14 adult male HSP/IgAV patients with cardiac involvement whose onset ages were between 19 and 71 years old. For these patients, 11 patients (78.5%) had renal involvement, and 12 had a clear therapeutic regimen. We also found that after treatment, 3 patients (21.4%) died, and 2 patient's prognoses were unknown (Table 3).

**Table 4:** Pediatric patients with HSP/IgAV accompanied by cardiac involvement in the literature.

**Patients Sex/Age (y)**	**Cardiac involvement**			**Other organs involvement**	**Treatments**	**Outcomes**	
	**Cardiac Symptoms/ Signs**	**ECG/Echocardiogram/MRI Findings**	**Other findings**			**Cardiac**	**Other organs**
Our patient, M/6	Chest tightness, crushing pain in precordial area	Coronary artery aneurysm (Echocardiogram)	CK/CK-MB?troponin I elevation	Kidney, Liver	MMF, MP/ Prednisone, Aspirin, Warfarin	Coronary arteries aneurysm (22 months)	Proteinuria disappeared; liver function normal (22 months)
M/9 Bloom et al. (22)	No	The left main and left anterior descending coronary artery dilation (Echocardiogram)	No	No	Aspirin, IVIG, Infliximab	Complete resolution (3 months)	**__**
M/3 Veetil et al. (23)	Persistent tachycardia	Periluminal coronary artery thickening (Echocardiogram)	No	No	Prednisone, IVIG	Complete resolution (1 month)	**__**
M/17 Zaidi et al. (24)	Shortness of breath and chest pain	Left ventricle dilatation and prominent coronary arteries (Echocardiogram)	No	Kidney, Liver, Pancreas	MP/ Prednisone, ACEI	Unknow	Renal function normal, proteinuria decreased; liver and pancreas unkown (3 months)
F/8 Yilmaz et al. (25)	Tachycardia	Pericardial effusion, right atrium thrombus, myocarditis (Echocardiogram and MRI)	BNP elevation	Kidney	Hemodialysis, MP/Prednisolone, CTX, AZA, ACEI	Complete resolution (8 months)	Kidney function normal, proteinuria decreased (8 months)
M/8 Cimaz et al. (26)	Cardiac murmur	Pericardial effusion; inversion T waves with repolarization abnormalities (Echocardiogram and ECG)	No	No	Symptomatic treatment	Complete resolution (a few weeks)	**__**
F/10 James et al. (27)	Apical III/VI holosystolic murmur	Severe mitral regurgitation, left atrium dilatation, diastolic dysfunction, mitral valve prolapses (Echocardiogram)	BNP elevation	Pulmonary hemorrhage	Mechanical ventilation, IVIG, Prednisone, ACEI	Mitral regurgitation (12 months)	Asymptomatic (12 months)
M/16 Shah and Hata (28)	Palpitations	Arrhythmia (ECG)	The troponins elevation	Kidney, CNS, GI bleed	MP/ Prednisone, Labetalol, Amiodarone, Metoprolol, CTX, MMF, ACEI	Complete resolution (9 months)	No recurrence of GI and neurological complications; kidney function normal, proteinuria decreased (9 months)
M/12 Migita et al. (29)	Carotid artery engorgement, hypotension, and narrow pulse pressure	Pericardial tamponade (Echocardiogram)	No	Kidney, small intestine, nervous system	Prednisone/MP, Surgery, Pericardiocentesis	Unknow	Unknow
M/11 Lecutier (30)	Systolic apical murmur	No	Myocardium necrosis with calcification (Necropsy)	Kidney	Unknow	Death (1 month)	Death (1 month)

*M, male; F, female; Electrocardiogram, ECG; MRI, Magnetic Resonance Imaging; BNP, Brain natriuretic peptide; MMF, Mycophenolate mofetil; CTX, Cyclophosphamide; MP, Methylprednisolone; AZA, azathioprine; CNS, Central Nervous System; ACEI, Angiotensin Converting Enzyme Inhibitor; GI, Gastrointestinal*.

## Discussion

Typical HSP/IgAV often involves the skin, gastrointestinal tract, joints, and kidneys. Cardiac involvement is an extremely rare complication of HSP/IgAV, with various manifestations. The earliest recorded case of HSP/IgAV complicated with cardiac involvement was an 11-year-old male patient reported by Lecutier in 1952, in which cardiac involvement manifested as myocardial necrosis, and the patient soon died (30). At present, only 24 cases of HSP/IgAV with definite cardiac involvement are recorded in the PubMed database, and 4 of these patients died. Therefore, when HSP/IgAV patients are afflicted with rare complications, lack of adequate attention may lead to poor prognoses. Further analysis of the reported cases of HSP/IgAV complicated with cardiac involvement revealed that compared with children, adults with HSP/IgAV complicated with cardiac involvement are more likely to have a poor prognosis and may need more active treatment. However, in this present study, we only carried out a simple analysis of previous case reports that lacks the support of large amount of data.

Coronary artery dilatation is common in patients with vasculitis such as Kawasaki disease (KD) and is the main cause of acquired heart disease in children. Previous studies found that about 55% of KD patients with coronary artery dilatation gradually improved over time (31). However, in recent years, there have been different opinions. Lin et al. (32) studied 1,073 KD patients and found that 40.6% of them had coronary artery lesions in the acute fever period. Further analysis of their prognoses found that KD patients with large coronary aneurysm had long-term coronary artery dilation that could lead to myocardial ischemia or sudden death (32). In our case, the child also had HSP/IgAV complicated with a large coronary artery aneurysm, and the coronary artery aneurysm did not improve after 22 months of follow-up treatment. This may indicate a poor long-term prognosis for the child, just like Kawasaki disease combined with coronary artery aneurysm. HSP/IgAV belongs to small vasculitis but rarely involves the coronary artery. However, current research has suggested that KD vasculitis and HSP/IgAV may have a common pathological mechanism (33). Noval et al. (33) established a KD mouse model and found IgA-complement component 3 (C3) immune complex deposition in cardiac lesions and kidneys, suggesting that KD vasculitis may be a form of IgA vasculitis. Kereiakes et al. (20) also found IgA and C3 deposits in the myocardial vascular wall through myocardial tissue biopsy of male patients with HSP, similar to IgAV. These findings may explain why HSP/IgAV that presents as small vasculitis can still involve the coronary arteries and even manifest as coronary dilation similar to KD. However, its exact pathogenesis needs further research. In recent years, other rheumatic diseases such as juvenile idiopathic arthritis (sJIA) and systemic lupus erythematosus (SLE) have also been reported to be complicated with coronary artery dilatation (34, 35). In addition, there are some reports of coronary artery dilatation in patients with infectious diseases such as EBV virus infection and COVID-19 (36, 37). It indicates that coronary artery dilatation may be more common than previously recognized. Unfortunately, this case did not carry out COVID-19 testing at the beginning of the disease, which is also one of the limitations of this article.

At present, the clinical treatment of HSP/IgAV combined with coronary artery dilatation is still derived from the empirical treatment of KD combined with coronary artery dilatation. Only a one case, a 9-year-old male child with HSP/IgAV with coronary artery dilatation who had left main coronary artery and anterior descending branch dilatation, can has been recorded in the PubMed database so far, reported by Bloom et al. (22). After IVIG, infliximab, and aspirin treatment, this patient's coronary artery returned to normal after 3 months (22). Unlike the previously reported cases, our patient is the only one known who had renal involvement and a coronary artery aneurysm at the same time. After treatment with glucocorticoid at the initial stage of onset, nephritis, coronary artery dilatation, and myocardial injury still occurred in this patient. Importantly, after treatment with aspirin and mycophenolate mofetil, renal injury gradually improved, but coronary artery dilatation continued to worsen, indicating that glucocorticoid may have a poor effect on preventing renal and cardiac involvement. From the treatment response of the child, we conclude that glucocorticoid combined with mycophenolate mofetil has a superior therapeutic effect on HSPN//IgAVN but a poor effect on coronary artery dilatation.

In summary, HSP/IgAV is the most common form of systemic vasculitis in children. It is also a self-limiting disease that often involves the skin, joints, gastrointestinal tract, and kidneys. The majority of children have a good prognosis. When HSP/IgAV is complicated with severe renal injury, the glucocorticoid combined with immunosuppressant therapy is effective. However, when HSP/IgAV is complicated with other rare complications such as coronary artery dilatation, glucocorticoid combined with immunosuppressant therapy is not very promising. Early administration of biological agents or IVIG may therefore be an acceptable choice. Unfortunately, the child in this report was not treated with biological agents or IVIG.

## Data Availability Statement

The original contributions presented in the study are included in the article/supplementary material, further inquiries can be directed to the corresponding author/s.

## Ethics Statement

The studies involving human participants were reviewed and approved by the Institutional Review Board of the Hunan Children's Hospital. Written informed consent to participate in this study was provided by the participants' legal guardian/next of kin. Written informed consent was obtained from the minor(s)' legal guardian/next of kin for the publication of any potentially identifiable images or data included in this article.

## Author Contributions

ZK, WW, MX, YD, and ZL contributed to conception and design of the study. WW, MX, and YD organized the data and follow-up. ZK wrote the first draft of the manuscript. ZL reviewed sections of the manuscript. All authors contributed to manuscript revision, read, and approved the submitted version.

## Funding

This work was supported by the Natural Science Fund of Hunan Province (2021JJ40269) and Scientific Research Key Project of Hunan Provincial Health Commission (202106012359).

## Conflict of Interest

The authors declare that the research was conducted in the absence of any commercial or financial relationships that could be construed as a potential conflict of interest.

## Publisher's Note

All claims expressed in this article are solely those of the authors and do not necessarily represent those of their affiliated organizations, or those of the publisher, the editors and the reviewers. Any product that may be evaluated in this article, or claim that may be made by its manufacturer, is not guaranteed or endorsed by the publisher.
